# Pharmacokinetics and clinical outcomes of low-dose nivolumab relative to conventional dose in patients with advanced cancer

**DOI:** 10.1007/s00280-024-04697-x

**Published:** 2024-07-26

**Authors:** Khushboo A. Gandhi, Aditi Shirsat, Sharat Kumar HJ, Ashish Chavan, Parnika Dicholkar, Saniya Shah, Nandini Menon, Vanita Noronha, Amit Joshi, Kumar Prabhash, Vijay Patil, Vikram Gota

**Affiliations:** 1 Department of Clinical Pharmacology, Advanced Centre for Treatment, Research and Education in Cancer (ACTREC), Tata Memorial Centre (TMC) Sector- 22, Kharghar, Navi Mumbai, 410210 India; 2 Department of Medical Oncology, Tata Memorial Hospital, Parel, Mumbai, 400012 India; 3 Homi bhabha National Institute, Mumbai, 400094 India; 4 Department of Medical Oncology, Advanced Centre for Treatment, Research and Education in Cancer (ACTREC), Tata Memorial Centre (TMC), Kharghar, Navi Mumbai, 410210 India

**Keywords:** Conventional dose, Low-dose, Nivolumab, Pharmacokinetics, Response, Toxicity

## Abstract

**Purpose:**

Nivolumab is approved at various doses, including 3 mg/kg, 240 mg and 480 mg flat doses at various dosing intervals. The concept of low-dose immunotherapy is gaining traction in recent years. However, there is a need to better understand the pharmacokinetics and clinical outcomes at lower doses.

**Methods:**

Patients were either administered 40 mg flat dose or 3 mg/kg Q2W/Q3W, depending on affordability as per prevailing hospital practice. All patients were hospitalized on day 1 and pharmacokinetic samples were collected at 0, 0.5, 1.0, 6.0, 24.0, 72.0 h and day 14 following administration of the first dose of nivolumab. Plasma nivolumab levels were measured by ELISA. Patients were followed up for response and toxicity.

**Results:**

Twenty five patients were included in the study. Fourteen received nivolumab at conventional dose (3 mg/kg), while 11 patients received low-dose (40 mg flat). The geometric means of dose normalized C_max_ and AUC_0-t_ were comparable between those who received conventional dose and low-dose of nivolumab (0.28 versus 0.23 µg/mL/mg and 0.0014 versus 0.0011 d/mL respectively). Nineteen patients were evaluable for response. ORR among patients who received conventional dose was 5/11 (45.5%) whereas it was 4/9 (44.4%) in the low-dose cohort. All 14 (100%) patients in conventional dosing group and 7/11 patients (63.64%) in low-dose group had treatment emergent adverse events. Grade ≥ 3 toxicities were observed in 4/14 patients in conventional dose group and none in low-dose group.

**Conclusion:**

Low-dose nivolumab leads to lower exposure in patients as compared with conventional dose, but low-dose was better tolerated, while response rates were comparable to conventional dose.

**Supplementary Information:**

The online version contains supplementary material available at 10.1007/s00280-024-04697-x.

## Introduction

In 2016, the United States Food and Drug Administration (FDA) approved Opdivo (nivolumab) immunotherapy as a treatment for patients with recurrent or metastatic head and neck squamous cell carcinoma (HNSCC) with disease progression on or after platinum-based therapy based on evidence from the Checkmate 141 randomized phase 3 trial. Checkmate 141 study reported nivolumab as the first single agent therapy to demonstrate survival benefit when used as a second line treatment in recurrent/metastatic HNSCC [1].

Nivolumab has demonstrated safety profiles up to doses of 10 mg/kg in various solid malignancies, initially approved at 3 mg/kg intravenously every two weeks (Q2W) by regulatory authorities across multiple tumor types [2, 3]. Subsequently, FDA approved use of nivolumab at a flat dose of 240 mg Q2W, followed by a flat dose of 480 mg every four weeks (Q4W) for advanced malignancies. This regulatory shift was based on comparable pharmacokinetic exposure, safety, and efficacy relative to the 3 mg/kg dose, resulting in the replacement of the 3 mg/kg dose across all monotherapy indications [4, 5]. Various studies have substantiated effectiveness of nivolumab at lower doses through pharmacodynamics modelling and receptor occupancy assays in peripheral blood, showing 70–90% receptor binding at ultralow doses as modest as 0.1–0.3 mg/kg, underscoring the antibody’s notable affinity and avidity [2, 6, 7]. Further, studies by Brahmer et al. showed that receptor occupancy of PD-1 exceeding 70% on circulating T cells can endure for at least two months. Similarly, recent research has documented sustained receptor occupancy of PD-1 for up to 90 days with a flat dose of 480 mg/960 mg and up to 78 days for a 20 mg flat dose, fostering interest in low-dose regimens [7, 8].

In early-stage and locally advanced stage, head and neck cancer (HCN) are typically treated with a curative intent; platinum, 5-fluorouracil (5FU), and cetuximab have traditionally been first-line treatments based on data from the phase III EXTREME trial for HCN [9]. More recently, nivolumab or pembrolizumab in the platinum refractory setting has become the recommended category 1 regimens as per National Comprehensive Cancer Network (NCCN) and European Society for Medical Oncology (ESMO) guidelines [10–12]. Despite being endorsed as a category 1 regimen in platinum refractory HCN by various healthcare agencies, the widespread use of nivolumab is hindered by its high cost. The estimated cost of nivolumab immunotherapy at the recommended standard dose of 3 mg/kg is approximately INR 2,75,000 per month for a single agent nivolumab regimen, rendering it financially prohibitive for over 90% of patients [13]. Even in developed countries like England, where healthcare coverage is provided, recent guidelines show reluctance to support nivolumab use in HCN due to its high cost. In such circumstances, low-dose nivolumab emerges as a plausible option for the management of advanced cancer, thereby broadening its accessibility and affordability. To overcome the financial challenges associated with the full dose of nivolumab in India, medical oncologists at Tata Memorial Hospital have adopted the practice of employing low-dose nivolumab. Notably, Patil et al. have demonstrated that supplementing metronomic therapy, comprising oral methotrexate at 9 mg/m^2^ once weekly, celecoxib at 200 mg twice daily, and erlotinib at 150 mg once daily, with a low-dose nivolumab of 20 mg flat dose every three weeks (Q3W) significantly improved 1-year Overall Survival (OS) from 16.3 to 43.4% (*P* = 0.0036) and increased median OS from 6.7 months to 10.1 months (*P* = 0.0052) for recurrent or newly diagnosed advanced HNSCC [13]. Moreover, their study showed that addition of low-dose nivolumab to metronomic therapy improved duration of response and PFS in HNSCC patients.

Furthermore, a pertinent case report by Abraham et al. highlight the efficacy of low-dose regimens like 40 mg Q2W of nivolumab in achieving a partial response without significant toxicity in a patient with sarcomatoid carcinoma of the right buccal mucosa [14]. Given the constraints and the demonstrated efficacy of low-dose nivolumab in various settings, it is crucial to comprehensively understand and compare the exposure (area under the concentration vs. time curve) following conventional and low doses.

Consequently, this study aims to assess the first-dose pharmacokinetics of nivolumab in Indian patients with advanced solid tumors, comparing conventional dose (3 mg/kg) and low-dose (40 mg flat). This study also seeks to elucidate the dose-exposure and dose-toxicity relationship, with outcomes expected to facilitate the optimization strategies for low-dose nivolumab use, especially in low or middle income country (LMIC), where affordability and accessibility are critical considerations in healthcare decisions.

## Patients and methods

### Study population

Patients with malignant solid tumors who were prescribed nivolumab were enrolled on this study. Patients with histologically or cytologically confirmed malignant solid tumors having at least one measurable disease as per Response Evaluation Criteria for Solid Tumors (RECIST) version 1.1 were enrolled. Other inclusion criteria included adequate hematological parameters (hemoglobin ≥ 9 g/dl, neutrophil > 1,500/per µL, platelets > 100,000/per µL), hepatic function within normal limits (bilirubin ≤ 1.5 times the upper limit of normal and, AST and ALT < 3 times the upper limit of normal) and renal function (creatinine clearance > 40 ml/min) with an ECOG PS of 0–1, and a life expectancy of ≥ 3 months. Exclusion criteria were the presence of multiple primary cancers, uncontrolled brain metastases, any active known or suspected autoimmune disease and history of any chronic or recurrent autoimmune disease, severe hypersensitivity, HIV infection and positive serology for HBV, HCV, pregnancy or lactation. Patients receiving any other anticancer therapy, having adverse drug reactions associated with prior treatments or surgical therapy with a possible influence on the study outcome and those requiring systemic treatment with either corticosteroids or other immunosuppressive medications within 14 days of study drug administration were also excluded.

### Study design

This study was conducted as a prospective, single-center, single-dose, pharmacokinetic (PK) study. Patients prescribed nivolumab at varying doses (3 mg/kg or 40 mg flat Q2W/Q3W) based on the discretion of the treating oncologist were screened and enrolled. All patients received intravenous nivolumab administered as a 60 min continuous infusion. Treatment with nivolumab continued until disease progression, the occurrence of intolerable adverse events, or the patient’s voluntary refusal of the drug for any reason. Adjustment to the nivolumab dose were made based on tolerability in subsequent cycles as per standard hospital practice. Response to treatment was systematically evaluated using PET CT scans at the end of every four cycles, and additionally, as clinically indicated thereafter. The assessment of response was done using RECIST criteria v1.1 with a follow-up evaluation at the three-month mark.

#### Pharmacokinetic sampling

A comprehensive analysis of the pharmacokinetic profile of a single dose of nivolumab was carried out in the present study. Patients were hospitalized on the first day of commencing treatment. Blood samples were collected from the patients at predetermined time intervals, including 0, 0.5, 1, 6, 24, 72 h and on day 14 after the administration of the first dose of nivolumab to measure plasma levels of nivolumab. The samples were collected in EDTA tubes and subsequently centrifuged at 3000 rpm for 10 min. The resulting supernatant plasma was separated and preserved in pre-labeled 1.7-ml micro centrifuge tubes at − 80 °C. Subsequently, the samples were analyzed within three month period from the date of collection. The bioanalysis of the samples was performed using Nivolumab (Opdivo^®^) (Human) ELISA Kit (ELISA Genie, Dublin, Ireland). The analysis was performed in accordance with the manufacturer’s protocol, with a standard range of 0–30 µg/mL for nivolumab concentration.

### Pharmacokinetic analysis

The pharmacokinetic parameters including area under the concentration vs. time curve from time zero to infinity (AUC_0-inf_), maximum (or peak) plasma concentration (C_max_), volume of distribution (Vd), clearance (Cl), and half-life (t_1/2_) of nivolumab were estimated using non-compartmental analysis (NCA) in Pumas v1.1.0 (Pumas-AI Inc., Baltimore, MD). This analytical approach allowed for a comprehensive evaluation of the drug’s pharmacokinetic behavior, providing insights into key parameters relevant to understanding its disposition and efficacy in the studied patient population.

## Safety and efficacy assessments

All adverse events were graded in accordance with the Common Terminology Criteria for Adverse Events (CTCAE) version 5.0. All response assessments were systematically complied until disease progression. The best response for each patient was categorically determined as either a complete response (CR), partial response (PR), stable disease (SD), or progressive disease (PD) as per RECIST 1.1. This standardized approach ensured consistent and comparable evaluation of both adverse events and treatment responses across the patient cohort.

### Statistical analysis

Continuous data in this study were expressed as either geometric mean or median (range), depending on the distribution of the data. Categorical data were expressed as frequency and percentage. To compare the pharmacokinetic parameters between the two studied doses, an unpaired student t-test was employed. Progression-free survival (PFS) (defined as the time from the start of nivolumab treatment to either disease progression or death due to any cause) was analyzed using the Kaplan–Meier method. The log-rank test was used to compare the PFS between the two studied doses. All computational analysis was performed using SPSS Version 23 and GraphPad Prism 8.0.2, ensuring robust and comprehensive evaluation of both continuous and categorical data, as well as survival outcomes in the context of nivolumab dosing regimens.

## Results

**Fig. 1:**
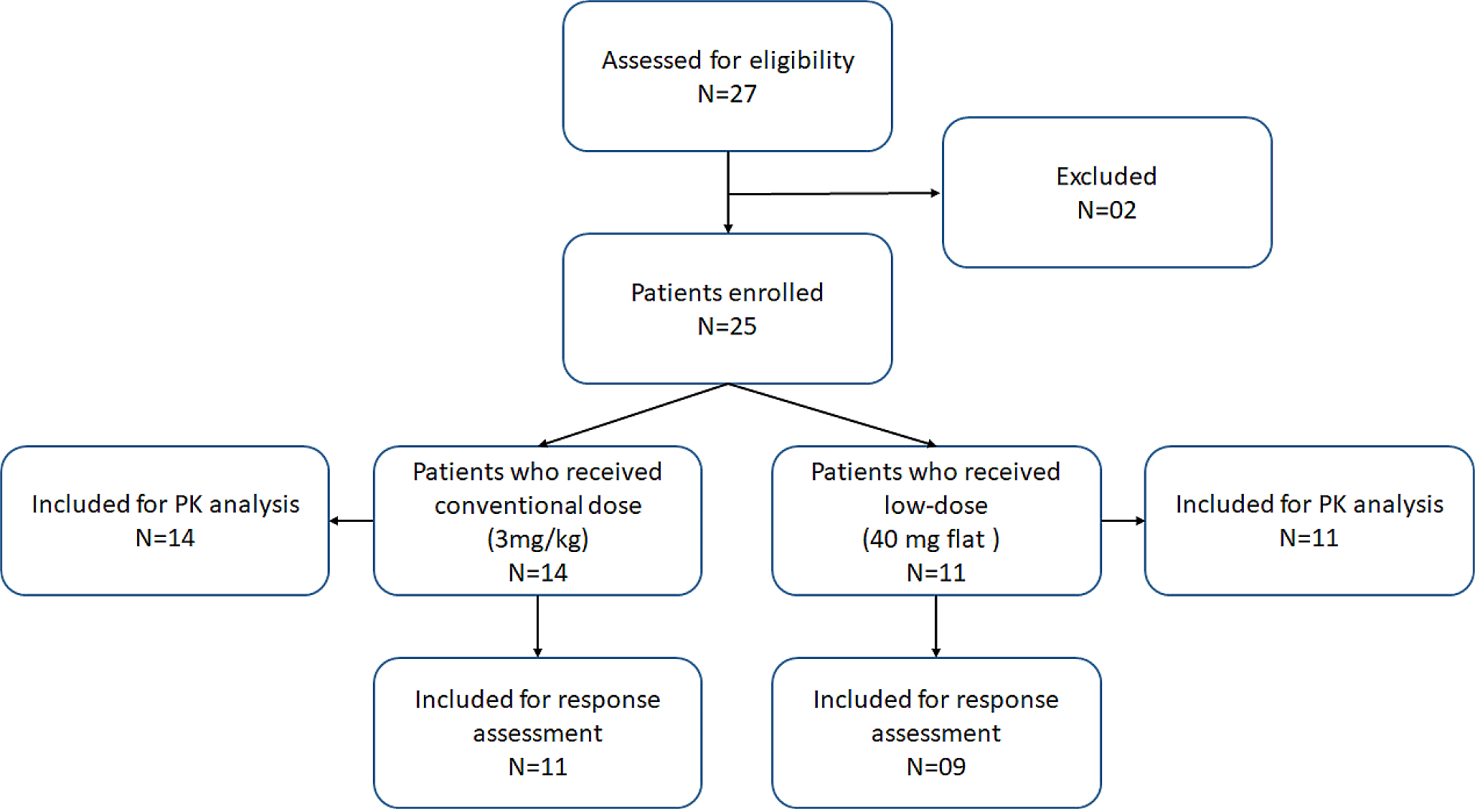
Schematic representation of study participants

**Patient Characteristics** Cancer patients with various histology for whom nivolumab was indicated as treatment were enrolled in the present study from 2019 to 2021. A total of 27 patients were enrolled in the study, of which two patients were screened failed. Consequently, the final analysis included a total of 25 patients. Among them, 14 patients received conventional dose (3 mg/kg Q2/3 W) of nivolumab, while the remaining 11 patients were administered low-dose (40 mg flat Q2/3 W) nivolumab (Fig. 1).

For patients receiving conventional dose of nivolumab, the median age was 49.5 years (range: 31–71) comprising 12 males and two females. On the other hand, patients receiving low-dose nivolumab had a median age of 54 years (range: 37–75) years with 11 males. The median of actual dose received by patients in conventional dose group was 170 (140–240) mg. A comprehensive overview of the patient’s baseline characteristics, covering ECOG performance and cancer type, is presented in Table 1. Further details regarding baseline characteristics of patients receiving conventional and low-dose nivolumab are described in supplementary Table 1.

**Table 1:** Baseline characteristics

Variable	All patients (*N*= 25)
Age (years) Median (range)	51 (31–75)
BSA (m^2^) Median (range)	1.62 (1.38–1.99)
Sex	
Female	2 (8%)
Male	23 (92%)
ECOG-PS	
0	2 (8%)
1	23 (92%)
Type of Cancer	
Head & neck cancer	22 (88%)
Thoracic cancer	2 (8%)
Urological cancer	1 (4%)
**Previous line of therapy received by patients**	**No. of patient**
0	8 (32%)
1	12 (48%)
2	3 (12%)
3	2 (8%)
S. Albumin (g/dL)	3.75 ± 0.46
S. Bilirubin (mg/dL)	0.52 ± 0.25
S. Creatinine (mg/dL)	0.69 ± 0.20
AST (U/L)	26.6 ± 9.31
ALT (U/L)	26.72 ± 17.68

### Pharmacokinetic analysis

**Fig. 2:**
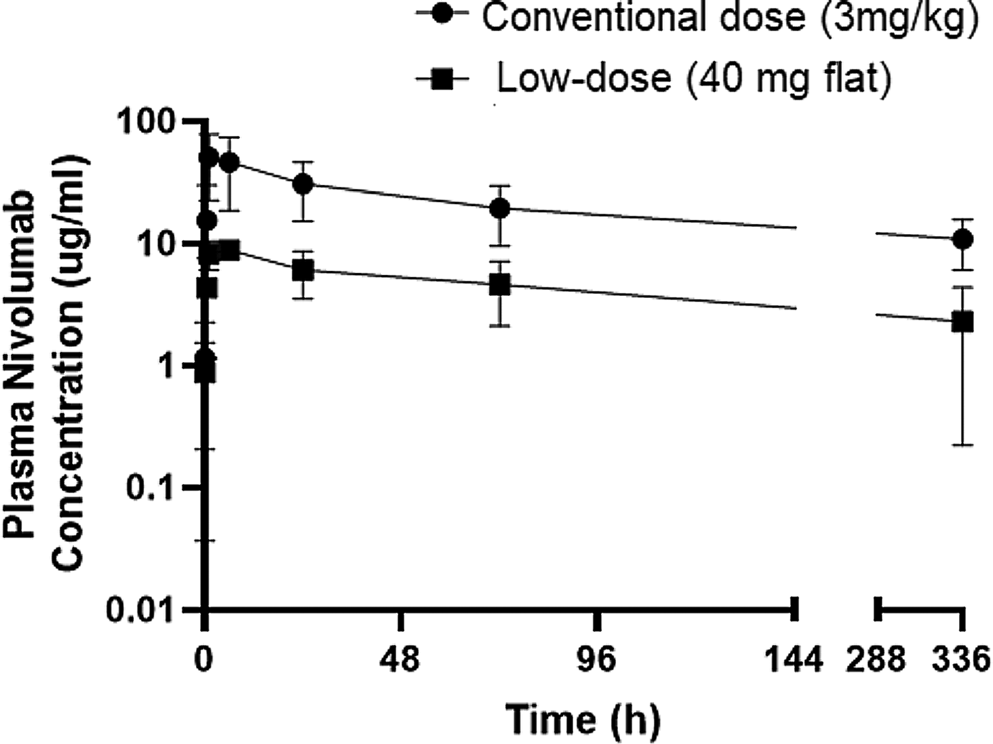
Log plasma nivolumab concentration versus time profiles post conventional dose (3 mg/kg) and low-dose (40 mg flat) administration of a single dose 60 min infusion of nivolumab

The pharmacokinetic parameters of nivolumab were estimated using the non-compartmental analysis method. The geometric means of plasma concentrations as a function of time following a 60 min intravenous infusion of a single dose of nivolumab, comparing both conventional and low-dose regimens are illustrated in Fig. 2. The geometric means of clearance (Cl) for patients receiving conventional dose and low-dose nivolumab was 393 mL/d and 417 mL/d, respectively. The geometric means of dose-normalized AUC_0-t_ were 0.0014 d/mL and 0.0011 d/mL for patients receiving conventional and low-dose nivolumab, respectively. A comprehensive summary of the first-dose pharmacokinetic parameters of nivolumab is provided in Table 2. Notably, half-life (t_1/2_), volume of distribution (V_d_), and clearance (Cl) were comparable for all the enrolled patients (*p* > 0.05). Furthermore, there were no statistically significant differences in dose-normalized C_max_ (*P* = 0.10) and AUC_0-t_ (*P* = 0.33) between patients who received conventional dose of nivolumab and those who received low-dose nivolumab.

**Table 2:** First-dose pharmacokinetic parameters of patients who received conventional dose (3 mg/kg) and low-dose (40 mg flat dose) of nivolumab

	Conventional Dose (3 mg/kg) (*N* = 14)				Low-dose (40 mg flat) (*N* = 11)			
	GeoMean	GeoCV%	Median	95% CI of GeoMean	GeoMean	GeoCV%	Median	95% CI of GeoMean
t_max_(d)			0.042 (0.042–0.25)				0.25 (0.021-1)	
c_max_(µg/mL)	47.3	61.3	42.8 (22.9–113)	34.12–65.50	9.06	16.3	8.75 (7.16–11.9)	8.13–10.10
c_max_/dose (µg*ml-1*mg-1)	0.28	53.8	0.26 (0.14–0.63)	0.20–0.37	0.23	16.3	0.22 (0.18–0.30)	0.20–0.25
t_1/2_(d)	11	60.8	11.9 (4.95–24.7)	7.92–15.14	11.8	92.2	9.76 (5.53–65.5)	6.11–22.71
vz (mL)	6211	49.6	7650 (3217–11,954)	4736–8144	7084	29.8	7599 (4708–9957)	5550–9040
Cl (mL/d)	393	38.2	425 (191–698)	317.6-486.5	417	117	507 (55.5–1026)	191.6-905.6
AUC_0_-_∞_((µg*d)/mL)	432	42.8	418 (229–941)	341.1-547.9	96	117	84.8 (39–721)	44.17–208.80
AUC_0_-_∞_/dose(µg*d/mL/mg)	0.0025	38.2	0.00236 (0.00143–0.00523)	0.0020–0.0031	0.0024	117	0.00212 (0.00097-0.018)	0.0011–0.0052
AUC_0 − t_((µg*d)/mL)	238	43.1	233 (131–521)	187.4-301.8	44.3	60	43 (12.9–97.5)	30.53–64.29
AUC_0 − t_/ dose(µg*d/mL/mg)	0.0014	33.8	0.00138 (0.00082–0.00289)	0.0011–0.0016	0.0011	60	0.00107 (0.00032–0.00244)	0.0007–0.0016

### Response

In this analysis, 20 out of the twenty-five patients (80%) enrolled in the study were included. Among them, 11 patients received conventional dose of nivolumab whereas nine patients received low-dose nivolumab. Notably, one patient (9.09%) who received conventional dose of nivolumab had a complete response. Further, one patient (9.09%) who received conventional dose and one patient (11.11%) who received low-dose nivolumab attained a partial response. At the three-month follow-up, disease progression was observed in 6 (54.5%) out of 11 patients who received conventional dose and in 5 (55.6%) out of 9 patients who received low-dose nivolumab. Furthermore, stable disease was reported in 3 (27.3%) out of 11 patients receiving the conventional dose and in 3 (33.3%) out of 9 patients receiving low-dose of nivolumab, as outlined in Table 3.

**Table 3:** Overall response evaluable in patients who received conventional dose (3 mg/kg) and low-dose (40 mg flat) of nivolumab at three months follow-up

	Conventional dose (3 mg/kg) (*N* = 11)		Low-dose (40 mg flat) (*N* = 09)	
	No. of patients	(%)	No. of patients	(%)
CR	01	9.1	00	0
PR	01	9.1	01	11.1
SD	03	27.8	03	33.3
PD	06	54.5	05	55.6

CR = complete response, PR = partial response, SD = stable disease, PD = progressive disease

### Progression free survival

**Fig. 3:**
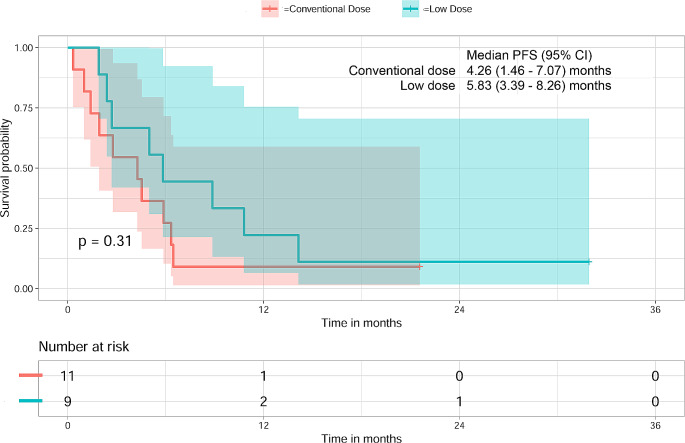
Kaplan-Meier survival curves illustrating the progression-free survival in patients who received conventional dose (3 mg/kg) and low-dose (40 mg flat) of nivolumab at three months follow-up. The median survival in patients who received conventional dose of nivolumab was 4.3 months with 95% CI of 1.46–7.07 months whereas for patient receiving low-dose nivolumab median PFS was 5.8 months with 95% CI of 3.39–8.26

The median PFS for patient receiving conventional dose of nivolumab was 4.3 months (1.46–7.07), while for patient receiving low-dose nivolumab, the median PFS was 5.8 months (3.39–8.26). Notably, there was no significant difference observed in the PFS of patients administered with either of the studied dose regimens of nivolumab (*P* = 0.31). The survival estimates for patients receiving both conventional and low-dose nivolumab are represented in Fig. 3. This data suggests comparable progression-free survival outcomes for patients treated with either the conventional or low-dose nivolumab regimens.

### Safety

Adverse events observed during the entire duration of nivolumab treatment are summarized in Table 4. All 14 (100%) who received conventional dose of nivolumab experienced adverse events of any grades, while only seven patients (63.64%) who received low dose of nivolumab reported adverse events of any grade. Among those who received conventional dose, four (28.57%) out of 14 patients had grade ≥ 3 immune-related adverse events, whereas, none of the patients who received low-dose nivolumab reported grade ≥ 3 immune-related adverse events. The grade ≥ 3 immune-related adverse events observed were fatigue, transaminitis, dysphagia and dermatitis. However, no cases of fatal toxicities were observed. The most common immune-related adverse events of any grade were fatigue and fever in both dosing groups (Table 4).

**Table 4:** Adverse events in patients who received conventional dose (3 mg/kg) and low-dose (40 mg flat dose) of nivolumab

	Conventional dose (3 mg/kg) (*N* = 14)				Low-dose (40 mg flat) (*N* = 11)			
	No. of patients with events (%)							
	Any grade		≥ Grade 3		Any grade		≥ Grade 3	
	**No.**	**%**	**No.**	**%**	**No.**	**%**	**No.**	**%**
Any adverse event	14	100	4	28.6	7	63.6	0	0
Immune-related adverse event	9	64.3	4	28.6	6	85.7	0	0
Fatigue*	7	50	1	7.1	3	27.3	0	0
Fever*	3	21.4	0	0	3	27.3	0	0
Anemia*	2	14.3	0	0	1	9.1	0	0
Leukopenia*	0	0	0	0	1	9.1	0	0
Leukocytosis*	0	0	0	0	1	9.1	0	0
Thrombocytopenia*	1	7.1	0	0	0	0	0	0
Hyperthyroidism*	0	0	0	0	1	9.1	0	0
Transaminitis*	1	7.1	1	7.1	1	9.1	0	0
Anorexia*	1	7.1	0	0	0	0	0	0
Nausea*	1	7.1	0	0	1	9.1	0	0
Dysphagia*	1	7.1	1	7.1	0	0	0	0
Dermatitis*	1	7.1	1	7.1	0	0	0	0
Oral mucositis*	1	7.1	0	0	0	0	0	0
Facial Edema	0	0	0	0	1	9.1	0	0
Seizures	1	7.1	0	0	0	0	0	0
Constipation	2	14.3	0	0	0	0	0	0
Hypochloraemia	1	7.1	0	0	0	0	0	0
Hypercalcemia	2	14.3	0	0	0	0	0	0
Eosinophilia	0	0	0	0	1	9.1	0	0
Hypokalemia	1	7.1	0	0	0	0	0	0
Hyperkalemia	1	7.1	0	0	0	0	0	0

*-Immune-related adverse events

### Exposure- toxicity relationship

**Fig. 4:**
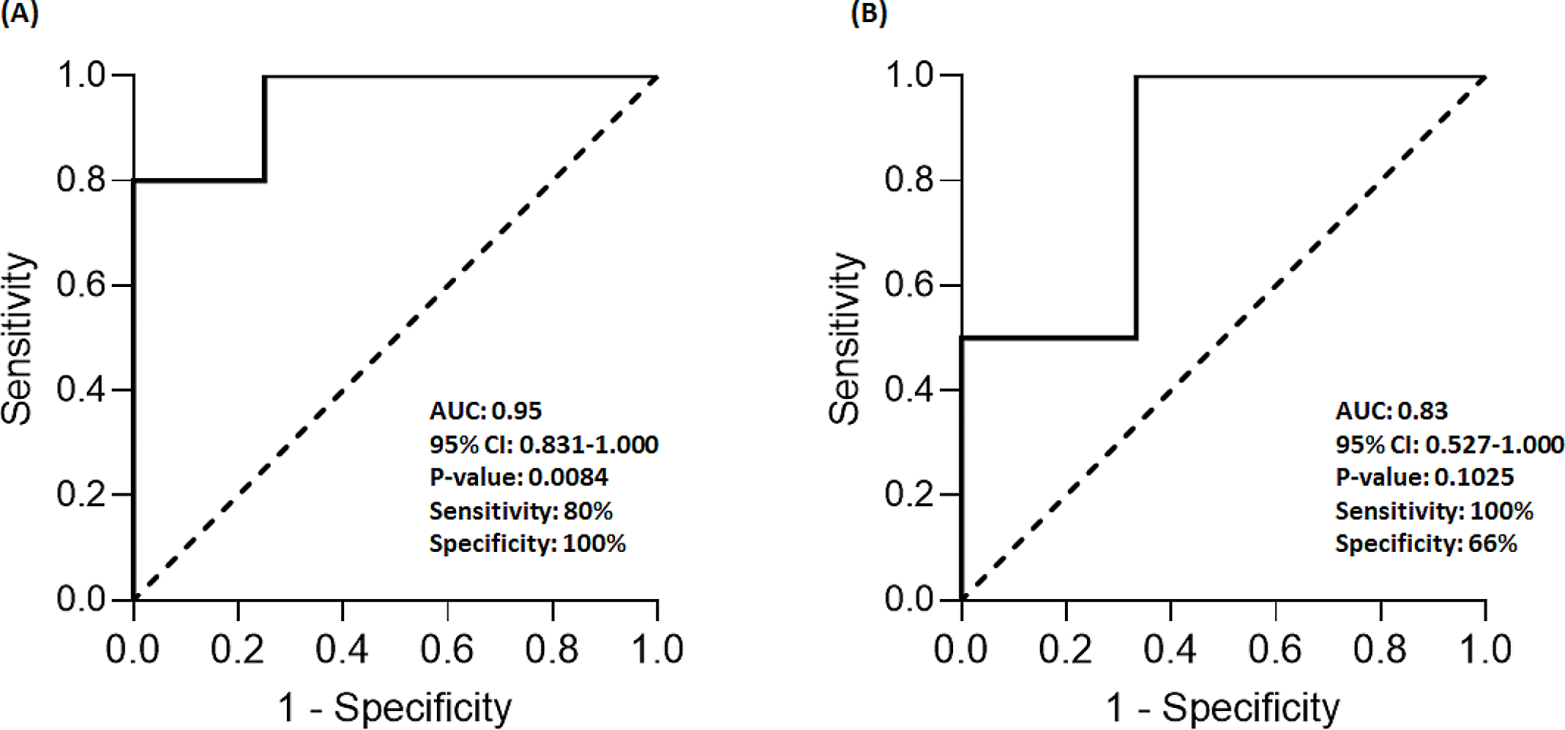
Receiver Operating Characteristic (ROC) curve to determine the optimal nivolumab AUC_0 − t_ level threshold for toxicity grade ≥ 2. (A) ROC for patients who received conventional dose (3 mg/kg) of nivolumab. The area under the curve and standard error was 0.95 (0.06). The most sensitive and specific toxicity limit would be an exposure of ≥ 2.69 d.µg/ml. for patients who received conventional dose of nivolumab. (B) ROC for patients who received low-dose (40 mg flat) of nivolumab. The area under the curve and standard error was 0.83 (0.15). ≥ No threshold could be established in patients receiving the low dose of nivolumab

Out of 25 patients, nivolumab treatment resulted in grade ≥ 3 adverse events in only four (28.57%) patients, all of whom received conventional dose of nivolumab. No relationship was observed between nivolumab AUC_0-t_ and the occurrence of grade ≥ 3 adverse events. Moreover, there was no treatment discontinuation due to adverse events of nivolumab in any of the patients. Additionally, the incidence of toxicity grade ≥ 2 and nivolumab exposure in patients receiving conventional dose and low-dose nivolumab was visualized in a Receiver Operating Characteristic (ROC) curve (Fig. 4). The threshold exposure that could discriminate between patients with and without grade ≥ 2 toxicity in conventional dose was found to be 2.69 d.µg/ml. No such threshold could be established in patients receiving low dose of nivolumab.

## Discussion

The present study investigated the pharmacokinetic profile and clinical outcomes of low-dose nivolumab (40 mg flat) compared to conventional dose (3 mg/kg) in a cohort of patients with heterogeneous solid tumors in real world scenario.

The pharmacokinetic analysis included measuring the geometric means of plasma nivolumab concentration–time profiles after the first dose of nivolumab at the two dose levels (Conventional dose vs. low-dose nivolumab). The pharmacokinetic analysis revealed that half-life (t_1/2_), volume of distribution (V_d_) and clearance (Cl) were comparable across all the enrolled patients irrespective of dose of nivolumab administered (*p* > 0.05). Also, there were no statistically significant differences in the dose normalized C_max_ (*P* = 0.10) and AUC_0-t_ (*P* = 0.33) between patients receiving conventional and low-dose nivolumab. This can be explained by linear pharmacokinetics of nivolumab in the range of 0.3–10 mg/kg with a dose-proportional increase in C_max_ and AUC_0-t_ and with low to moderate inter-individual variability observed at each dose level (CV = 7 to 45%) [15]. The derived and estimated pharmacokinetic parameters of nivolumab in the present study were consistent with the findings from previously reported pharmacokinetic studies [16].

Moreover, receptor occupancy assays in peripheral blood coupled with flow cytometry analysis, supported the use of low-dose nivolumab. The study indicated that doses as low as 0.1 mg/kg might maintain efficacy, given plateau receptor occupancy of PD-1 by nivolumab are achieved at this concentration [2, 6, 7]. This indicate high affinity/avidity of the clinical antibody. Additionally, the concentration of nivolumab achieved after administration of conventional dose is 47.3 µg/ml and 9.06 µg/ml after low-dose nivolumab whereas the required concentration for activity is 1.2 µg/ml [6]. The present study’s results are encouraging as they demonstrate that low-dose nivolumab can achieve therapeutic levels in cancer patients and these levels are comparable to those achieved with the conventional dosing regimen. This supports the potential feasibility and effectiveness of utilizing lower doses of nivolumab.

The median PFS (4.3 months) observed in patients who received conventional dose was comparable with those in CheckMate 141 study (2.0 months) [1]. Moreover, median PFS between conventional and low-dose nivolumab did not significantly differ, with both groups exhibiting clinically relevant outcomes (4.3 months (1.46–7.07) vs. 5.8 months (3.39–8.26) (*P* = 0.31), respectively). Similarly, the objective response rate (ORR) was 18.2% in conventional dose group and 11.1% in low-dose group, which was comparable between the two studied groups. The results are in accordance with a study by Yoo et al., who demonstrated no significant difference in PFS or OS or ORR between standard dose and low-dose nivolumab [17]. More recently, Patil et al. reported improved 1 year survival from 16.3 to 43.4% (*P* = 0.0036) and median OS 6.7 months to 10.1 months (*P* = 0 0.0052) by adding nivolumab at low-dose of 20 mg flat dose Q3W to metronomic therapy [13]. The observed median PFS in the low-dose nivolumab patients (5.8 months) were comparable with the PFS of patients (6.6 months) receiving 20 mg Q3W nivolumab in combination with metronomic therapy for HNSCC patients [13]. Lepik et al. also showed that in patients with relapsed or resistant Hodgkin lymphoma, the median PFS observed with a flat 40 mg Q2W dosage regimen was comparable to that of patients receiving a conventional dosage regimen (18.4 vs. 14.7 months) [18]. Interestingly, a number of studies have shown that ORR is correlated with nivolumab clearance rather than nivolumab dosage [2, 19]. In a similar vein, Mallardo et al. demonstrated correlation between week 12 nivolumab concentration and patients’ outcomes in terms of survival and tumor response [20]. We did not look into the correlation between clearance and outcomes because the dose of nivolumab administered to the patients was variable. However, our findings agree with the observation made by Lepik et al. in that outcomes with a lower flat dose was comparable to conventional dose. The geometric means of clearance of conventional and low-dose nivolumab (393 vs. 417 mL/d) did not differ significantly in the current trial, and the ORRs of the two dosing sets of nivolumab (2/11 vs. 1/9) were also similar.

The study reported all the adverse event observed throughout the study period. Adverse events of grade ≥ 3 occurred in 28.57% of the patients who received conventional dose versus 0% who received low-dose nivolumab. There was no treatment discontinuation due to adverse events of nivolumab in any of the patients. The study strongly indicates that low-dose nivolumab has lower toxicity as compared to conventional dose. Thus, this study showed that the safety is definitely better with low-dose as compared to conventional dose whereas efficacy is comparable between the two doses.

Considering the overall benefit imparted by nivolumab, it becomes imperative to explore various strategies that enable its inclusion in the treatment regimen of HNC while simultaneously reducing its cost making it available to larger population. In this context, the concept of low-dose nivolumab emerges as a potential solution, offering improved access and affordability of nivolumab in LMICs. Our study indicates that low-dose nivolumab is a practical approach, in metastatic/recurrent HNC in resource limited settings such as LMICs in real world scenario.

There are a few limitations of the study. This was a pilot study, and therefore the smaller sample size was included. As it was not powered for response and toxicity assessment, external validity of these findings is limited. The study population included patients with heterogeneous solid tumors. However, 22 out 25 patients were diagnosed with head and neck cancer. Since our goal was to compare the pharmacokinetics of conventional dose and low-dose nivolumab, early sampling method was employed, even though nivolumab has a longer half -life. Majority of the patients died or had progression of the disease before they received the 5th cycle of nivolumab. Hence, the steady state levels of nivolumab couldn’t be determined. Further studies can estimate this in indications which has allow steady state sampling.

To conclude, in this mixed cohort of patients, we report that low-dose nivolumab leads to lower exposure as compared with conventional dose of nivolumab. However, the dose normalized exposure of low-dose nivolumab was comparable to conventional dose indicating linear pharmacokinetics. Low-dose was better tolerated, and clinical outcomes were comparable to conventional-dose indicating that low-dose nivolumab does not compromise efficacy and improves safety. These findings call for head-to-head comparison of low-dose and conventional doses of nivolumab across therapy areas to improve access, affordability and safety of the immunotherapy regimen.

## Electronic supplementary material

Below is the link to the electronic supplementary material.

Supplementary Material 1

## Data Availability

No datasets were generated or analysed during the current study. **Code availability** Not applicable.
