# Supplementary material for: Pharmacokinetics and clinical outcomes of low-dose nivolumab relative to conventional dose in patients with advanced cancer
Source: Cancer Chemother Pharmacol. 2024 Jul 26;94(5):659–68. doi: 10.1007/s00280-024-04697-x (PMC11470857; doi:10.1007/s00280-024-04697-x)
Supplement: Supplementary file 1 — Supplementary Material 1 [file 280_2024_4697_MOESM1_ESM.docx]

**Supplementary table 1: Comparison of patient demographics between the two doses**

| **Variable** | **Conventional dose**  **(3 mg/kg)**  **(n = 14)** | **Low-dose**  **(40 mg flat)**  **(n = 11)** |
| --- | --- | --- |
| **Age (years) Median (range)** | 49.5 (31-71) | 54 (37-75) |
| **BSA (m^2^) Median (range)** | 1.58 (1.38-1.99) | 1.65 (1.47-1.79) |
| **Sex** | **No. (%)** | **No. (%)** |
| Female | 2 (14.28) | 0 |
| Male | 12 (85.71) | 11 (100) |
| **ECOG-PS** |  |  |
| 0 | 1 (7.14) | 1 (9.09) |
| 1 | 13 (92.85) | 10 (90.90) |
| **Dosing regimen** |  |  |
| Q2W | 12 (85.7) | 09 (81.8) |
| Q3W | 2 (14.3) | 2 (18.2) |
| **Type of Cancer** |  |  |
| Head & neck cancer | 14 (100) | 8 (72.72) |
| Thoracic cancer | 0 | 2 (18.18) |
| Urological cancer | 0 | 1 (9.09) |
| **Previous line of therapies** | **No. of patient received previous line of therapies (%)** | **No. of patient received previous line of therapies (%)** |
| 0 | 5 (35.7) | 3 (27.3) |
| 1 | 6 (42.8) | 6 (54.5) |
| 2 | 1 (7.1) | 2 (18.2) |
| 3 | 2 (14.3) | 0 |
| **S. Albumin (g/dL)** | 3.86 ±0.51 | 3.58 ±0.38 |
| **S. Bilirubin (mg/dL)** | 0.55 ±0.28 | 0.47 ±0.19 |
| **S. Creatinine (mg/dL)** | 0.67 ±0.23 | 0.70 ±0.15 |
| **AST (U/L)** | 24.78 ±8.14 | 27.2 ±9.40 |
| **ALT (U/L)** | 26.42 ±20.17 | 25.3 ±14.35 |
